# Report of an Operation

**Published:** 1894-04

**Authors:** S. E. Davenport

**Affiliations:** New York


### REPORT OF AN OPERATION.[1]

BY DR. S. E. DAVENPORT, NEW YORK.

DENTISTS are occasionally called upon to treat mouths in which, while there is no particular tendency towards pyorrhea alveolaris, certain teeth, usually the lower incisors, will be found very loose, the bony process much absorbed, and the gum thickened and spongy, with perhaps a slight exudation of pus.

This condition in such mouths is usually of local origin, caused by the encroachment of salivary calculus, the other teeth in the mouth being ordinarily in very good condition and of unquestioned longevity, and it always seems sad to be obliged to advise the extraction of these loose teeth, particularly as it is difficult to replace them with artificial substitutes which shall be both comfortable and natural.

I will confess that on several occasions I have seriously considered the advisability of in some way fastening such teeth securely to the firm ones of the same jaw, for the sake of saving the patient the annoyance of a plate, although recognizing at the same time the many objections to all such plans.

On one occasion the temptation to attempt the retention of such teeth was increased by the pleadings of the patient, a gentleman about seventy years of age, in good health and of good physique, who had more than the usual horror of artificial teeth, assuring me that he would bear any fatigue and put up with any discomfort if I would but save his natural teeth.

The teeth had as usual elongated considerably, and when the jaws were closed, the four inferior incisors would sometimes shut inside and sometimes outside the upper ones, though the roots were long and still had quite an attachment to the gum.

The first step was to grind down the elongated teeth with corundum stones until they would clear the upper ones when closed. This made the teeth very broad at the top, and enabled me to cut a rather deep longitudinal groove in the cutting-edges of not only the loose incisors, but also the firm cuspids on each side, into which groove a small twenty-karat gold wire was fitted, filling about one-half the calibre of the groove.

At the next sitting, the rubber dam being first adjusted, the gold wire was secured to its position in the groove with zinc phosphate,

---

[1] Read before the New York Odontological Society, January 10, 1894.

which, when fully hard, was cut away from two teeth at a time and replaced by semi-cohesive gold, malleted in, each tooth being held as firmly as possible with the thumb and fingers of the left hand while being worked upon. It was, of course, difficult to pack the gold as solidly around the wire in the loose teeth as in the two cuspids, but by looping the strips of gold over the wire from one side of the groove to the other, a very good adaptation was obtained, the fifteen-per-cent. platinized gold being used for the surface to give the greatest possible resistance to the forces of mastication.

The operation was successful in that the teeth were firmly held and made perfectly comfortable for the patient, who was instructed to use a small quill tooth-pick between the teeth after each meal, in addition to the usual thorough rinsing, so that the proximate surfaces might be kept clean.

At a subsequent sitting, the teeth were scaled and the gums treated, and within two weeks the discharge of pus had ceased, and the gums took on a healthy color and appearance.

This operation was performed in June, 1890, and was satisfactory in its results for just one year, when the gold around the wire in two of the incisors loosened. After new gold had been packed into those two teeth, I felt obliged to admit that the teeth could not be kept for any great length of time unless some additional protection could be given, as the strain from incising and masticating was too great for the strength which could be obtained.

An impression was accordingly taken, and upon the dies obtained a cap of pure gold, about No. 30, was swaged to fit the upper two-thirds of the lingual surfaces of the six teeth, and extending over the tops of the teeth to a point, perhaps, one-fourth of the distance down the labial surface.

This cap, being carefully fitted, was cemented to place, in June, 1891, with zinc phosphate, and the edges of the cap burnished to the teeth wherever possible.

This pure gold cap has become loose, I think, twice since it was first put on, but it is easily readjusted and has in every way fulfilled my expectations, for the teeth are preserved, and, I think, can be indefinitely.

Such a contrivance would be unsightly in a lady's mouth, but this gentleman, having a heavy moustache and beard, exhibits the golden line only when the joke is a particularly good one.

It would be a brave man indeed who would make a claim of originality for anything before this Society, and I will therefore say that I knew of the use by other dentists of this method for securing loose lower incisors years ago, so far as the gold wire and the strengthening fillings about it are concerned, but if the pure gold cap has been used by others I have never happened to hear of it.

